# Supplementary material for: A Likelihood Approach for Real-Time Calibration of Stochastic Compartmental Epidemic Models
Source: PLoS Comput Biol. 2017 Jan 17;13(1):e1005257. doi: 10.1371/journal.pcbi.1005257 (PMC5240920; doi:10.1371/journal.pcbi.1005257)
Supplement: S1 File — (TAR.GZ) [file pcbi.1005257.s014.tar.gz › HSPH_Online-SI-Revision/PF08/n10-extreme/n10-extreme_PF2_table4.pdf]

1	newly 3 weeks spec	135.	316.82	{290., 345.}	134.681	0.369157	newly 3 weeks cumul	629.	1266.45	{1209., 1313.}	101.343	0.303477	newly 5 weeks spec	61.	177.76	{158., 198.}	191.41	0.462393	newly 5 weeks cumul	793.	1685.12	{1631., 1743.}	112.499	0.326866	newly 54 weeks cumul	895.	2215.03	{2153., 2289.}	147.489	0.392912
2	newly 3 weeks spec	274.	348.67	{313., 388.}	27.3467	0.103397	newly 3 weeks cumul	1010.	1218.14	{1120., 1310.}	20.703	0.0809273	newly 5 weeks spec	172.	238.85	{208., 284.}	38.8663	0.140008	newly 5 weeks cumul	1394.	1750.13	{1620., 1890.}	25.5473	0.0978588	newly 50 weeks cumul	1905.	2815.12	{2568., 3175.}	47.7753	0.168036
3	newly 3 weeks spec	87.	237.98	{218., 258.}	173.54	0.436095	newly 3 weeks cumul	473.	1003.05	{971., 1036.}	112.061	0.326273	newly 5 weeks spec	29.	125.61	{110., 142.}	333.138	0.634206	newly 5 weeks cumul	554.	1303.18	{1256., 1336.}	135.231	0.371314	newly 56 weeks cumul	592.	1630.06	{1570., 1677.}	175.348	0.439602
4	newly 3 weeks spec	199.	274.15	{230., 312.}	37.8241	0.136601	newly 3 weeks cumul	783.	1018.35	{904., 1116.}	30.0958	0.112817	newly 5 weeks spec	95.	179.49	{147., 207.}	88.9368	0.272552	newly 5 weeks cumul	1007.	1423.02	{1256., 1569.}	41.3128	0.1485	newly 50 weeks cumul	1308.	2112.52	{1751., 2396.}	61.5076	0.205269
5	newly 3 weeks spec	213.	278.18	{246., 309.}	30.6009	0.114284	newly 3 weeks cumul	916.	1103.22	{1011., 1214.}	20.4389	0.079854	newly 5 weeks spec	132.	163.46	{142., 186.}	24.1667	0.0921751	newly 5 weeks cumul	1203.	1479.83	{1348., 1633.}	23.0116	0.0890015	newly 53 weeks cumul	1516.	1973.34	{1772., 2183.}	30.1675	0.11343
6	newly 3 weeks spec	232.	342.31	{304., 377.}	47.5474	0.16736	newly 3 weeks cumul	932.	1334.71	{1197., 1444.}	43.2092	0.154944	newly 5 weeks spec	93.	200.07	{176., 225.}	115.129	0.330771	newly 5 weeks cumul	1166.	1796.55	{1630., 1939.}	54.078	0.186739	newly 54 weeks cumul	1415.	2419.36	{2178., 2608.}	70.9795	0.231933
7	newly 3 weeks spec	196.	339.17	{287., 381.}	73.0459	0.235704	newly 3 weeks cumul	905.	1329.49	{1216., 1467.}	46.905	0.16576	newly 5 weeks spec	97.	196.49	{159., 228.}	102.567	0.302138	newly 5 weeks cumul	1165.	1787.02	{1609., 1967.}	53.3923	0.184257	newly 55 weeks cumul	1418.	2384.95	{2058., 2672.}	68.1911	0.22334
8	newly 3 weeks spec	285.	331.02	{305., 356.}	16.2737	0.0647481	newly 3 weeks cumul	1128.	1240.68	{1158., 1293.}	10.1046	0.0415056	newly 5 weeks spec	172.	206.39	{185., 229.}	20.4942	0.0797132	newly 5 weeks cumul	1506.	1709.03	{1594., 1779.}	13.5212	0.0547015	newly 52 weeks cumul	1911.	2434.94	{2228., 2558.}	27.4171	0.104642
9	newly 3 weeks spec	224.	308.32	{272., 340.}	37.6429	0.137036	newly 3 weeks cumul	973.	1176.75	{1071., 1262.}	20.9404	0.0818485	newly 5 weeks spec	131.	190.15	{163., 215.}	45.1527	0.159379	newly 5 weeks cumul	1278.	1611.09	{1452., 1717.}	26.0634	0.0997869	newly 52 weeks cumul	1632.	2268.98	{2008., 2441.}	39.0306	0.141915
10	newly 3 weeks spec	311.	354.36	{314., 394.}	14.7974	0.0588849	newly 3 weeks cumul	1154.	1306.65	{1229., 1403.}	13.4983	0.05437	newly 5 weeks spec	185.	224.44	{197., 258.}	21.4486	0.0819797	newly 5 weeks cumul	1603.	1813.83	{1710., 1957.}	13.3543	0.0536789	newly 52 weeks cumul	2165.	2619.29	{2400., 2867.}	21.0397	0.0815753
11	newly 3 weeks spec	280.	322.84	{294., 344.}	15.3643	0.0613074	newly 3 weeks cumul	1095.	1131.63	{1100., 1172.}	3.72146	0.0157997	newly 5 weeks spec	155.	227.65	{206., 246.}	46.871	0.165966	newly 5 weeks cumul	1475.	1632.59	{1588., 1685.}	10.6895	0.0439495	newly 47 weeks cumul	1957.	2671.97	{2538., 2775.}	36.534	0.134997
12	newly 3 weeks spec	241.	364.32	{326., 402.}	51.1701	0.178114	newly 3 weeks cumul	1116.	1348.53	{1274., 1436.}	20.836	0.0817185	newly 5 weeks spec	113.	229.57	{202., 255.}	103.159	0.30596	newly 5 weeks cumul	1420.	1870.58	{1765., 1987.}	31.731	0.119128	newly 52 weeks cumul	1677.	2709.79	{2527., 2919.}	61.5856	0.207451
13	newly 3 weeks spec	180.	262.24	{234., 299.}	45.6889	0.161567	newly 3 weeks cumul	742.	948.04	{876., 1062.}	27.7682	0.10535	newly 5 weeks spec	114.	180.33	{157., 209.}	58.1842	0.196038	newly 5 weeks cumul	985.	1348.07	{1237., 1518.}	36.8599	0.135029	newly 48 weeks cumul	1277.	2104.9	{1884., 2420.}	64.8316	0.214968
14	newly 3 weeks spec	185.	279.32	{218., 327.}	50.9838	0.174329	newly 3 weeks cumul	825.	1085.05	{903., 1230.}	31.7976	0.117315	newly 5 weeks spec	104.	165.29	{124., 208.}	59.6058	0.19664	newly 5 weeks cumul	1066.	1466.68	{1172., 1688.}	37.8724	0.136417	newly 53 weeks cumul	1314.	1991.12	{1489., 2369.}	51.8676	0.176705
15	newly 3 weeks spec	149.	297.56	{266., 327.}	99.7047	0.298813	newly 3 weeks cumul	603.	1094.21	{1005., 1171.}	81.461	0.258068	newly 5 weeks spec	76.	195.61	{169., 221.}	157.382	0.407991	newly 5 weeks cumul	785.	1533.37	{1396., 1647.}	95.3338	0.289905	newly 50 weeks cumul	962.	2280.13	{2009., 2486.}	137.02	0.373212
16	newly 3 weeks spec	236.	348.63	{311., 383.}	47.7246	0.167866	newly 3 weeks cumul	954.	1354.65	{1273., 1457.}	41.9969	0.151639	newly 5 weeks spec	131.	202.4	{172., 234.}	54.5038	0.186256	newly 5 weeks cumul	1261.	1824.01	{1698., 1964.}	44.6479	0.159551	newly 53 weeks cumul	1555.	2464.52	{2245., 2724.}	58.49	0.198698
17	newly 3 weeks spec	218.	315.49	{269., 367.}	44.7661	0.157963	newly 3 weeks cumul	974.	1247.96	{1096., 1349.}	28.1273	0.106119	newly 5 weeks spec	107.	179.82	{148., 211.}	68.0561	0.221799	newly 5 weeks cumul	1233.	1668.19	{1444., 1853.}	35.2952	0.129567	newly 55 weeks cumul	1481.	2198.21	{1933., 2461.}	48.4274	0.169228
18	newly 3 weeks spec	202.	284.88	{256., 314.}	41.0297	0.147889	newly 3 weeks cumul	887.	1126.25	{1066., 1185.}	26.9729	0.103183	newly 5 weeks spec	107.	164.14	{144., 182.}	53.4019	0.183591	newly 5 weeks cumul	1132.	1507.54	{1417., 1588.}	33.1749	0.123855	newly 53 weeks cumul	1431.	2010.83	{1888., 2124.}	40.5192	0.147064
19	newly 3 weeks spec	155.	352.89	{310., 400.}	127.671	0.355506	newly 3 weeks cumul	791.	1413.97	{1300., 1606.}	78.7573	0.250887	newly 5 weeks spec	62.	199.63	{172., 227.}	221.984	0.505359	newly 5 weeks cumul	957.	1879.79	{1735., 2134.}	96.4253	0.291732	newly 55 weeks cumul	1067.	2466.85	{2221., 2857.}	131.195	0.362278
20	newly 3 weeks spec	277.	345.62	{308., 381.}	24.787	0.0947619	newly 3 weeks cumul	1115.	1308.3	{1213., 1381.}	17.3363	0.0688748	newly 5 weeks spec	154.	214.56	{189., 237.}	39.3247	0.142408	newly 5 weeks cumul	1485.	1797.24	{1668., 1905.}	21.0263	0.0823205	newly 52 weeks cumul	1930.	2541.6	{2321., 2742.}	31.6891	0.118691
21	newly 3 weeks spec	230.	305.89	{279., 330.}	32.9957	0.122727	newly 3 weeks cumul	931.	1103.56	{1018., 1160.}	18.5349	0.0731183	newly 5 weeks spec	137.	208.48	{185., 234.}	52.1752	0.180426	newly 5 weeks cumul	1250.	1566.35	{1420., 1656.}	25.308	0.0972752	newly 49 weeks cumul	1622.	2431.49	{2199., 2564.}	49.9069	0.174964
22	newly 3 weeks spec	126.	264.82	{240., 288.}	110.175	0.321128	newly 3 weeks cumul	596.	1122.1	{1061., 1201.}	88.2718	0.274226	newly 5 weeks spec	39.	136.	{115., 156.}	248.718	0.538435	newly 5 weeks cumul	702.	1448.88	{1364., 1547.}	106.393	0.313956	newly 56 weeks cumul	785.	1786.7	{1670., 1932.}	127.605	0.356021
23	newly 3 weeks spec	183.	334.65	{281., 396.}	82.8689	0.258965	newly 3 weeks cumul	736.	1204.15	{1064., 1379.}	63.6073	0.211833	newly 5 weeks spec	84.	224.84	{187., 265.}	167.667	0.423481	newly 5 weeks cumul	938.	1706.38	{1490., 1969.}	81.9168	0.257671	newly 51 weeks cumul	1140.	2610.7	{2249., 3053.}	129.009	0.35674
24	newly 3 weeks spec	218.	260.97	{234., 286.}	19.7844	0.0772054	newly 3 weeks cumul	857.	960.73	{906., 1018.}	12.5239	0.0509154	newly 5 weeks spec	150.	168.92	{148., 189.}	14.6	0.0585041	newly 5 weeks cumul	1186.	1340.85	{1250., 1407.}	13.5793	0.0550168	newly 49 weeks cumul	1579.	2001.43	{1872., 2110.}	26.8733	0.102644
25	newly 3 weeks spec	123.	317.37	{292., 342.}	158.024	0.410846	newly 3 weeks cumul	613.	1201.21	{1152., 1263.}	95.956	0.291864	newly 5 weeks spec	53.	193.04	{173., 214.}	264.226	0.559954	newly 5 weeks cumul	719.	1643.26	{1568., 1739.}	128.548	0.358704	newly 52 weeks cumul	805.	2315.53	{2224., 2436.}	187.643	0.458578
26	newly 3 weeks spec	82.	253.57	{206., 295.}	209.232	0.486424	newly 3 weeks cumul	370.	1037.79	{920., 1177.}	180.484	0.446018	newly 5 weeks spec	10.	142.14	{109., 175.}	1321.4	1.1455	newly 5 weeks cumul	409.	1370.98	{1209., 1572.}	235.203	0.522963	newly 55 weeks cumul	439.	1772.6	{1519., 2059.}	303.781	0.602572
27	newly 3 weeks spec	253.	344.62	{317., 375.}	36.2134	0.13317	newly 3 weeks cumul	1055.	1338.76	{1281., 1408.}	26.8967	0.103111	newly 5 weeks spec	136.	202.9	{181., 228.}	49.1912	0.171895	newly 5 weeks cumul	1391.	1808.3	{1726., 1931.}	30.	0.113501	newly 53 weeks cumul	1730.	2466.82	{2324., 2669.}	42.5908	0.153367
28	newly 3 weeks spec	143.	267.37	{233., 311.}	86.972	0.269112	newly 3 weeks cumul	714.	1112.67	{1030., 1247.}	55.8361	0.191608	newly 5 weeks spec	59.	140.	{115., 176.}	137.288	0.370096	newly 5 weeks cumul	874.	1445.41	{1334., 1642.}	65.3787	0.21706	newly 56 weeks cumul	975.	1806.87	{1635., 2161.}	85.32	0.265496
29	newly 3 weeks spec	161.	304.1	{265., 341.}	88.882	0.274248	newly 3 weeks cumul	747.	1128.59	{1047., 1226.}	51.083	0.178301	newly 5 weeks spec	83.	192.5	{165., 219.}	131.928	0.362917	newly 5 weeks cumul	974.	1564.2	{1450., 1707.}	60.5955	0.204776	newly 51 weeks cumul	1110.	2257.82	{2034., 2554.}	103.407	0.306731
30	newly 3 weeks spec	187.	330.53	{290., 374.}	76.754	0.245499	newly 3 weeks cumul	802.	1212.25	{1109., 1372.}	51.1534	0.17826	newly 5 weeks spec	78.	212.35	{182., 246.}	172.244	0.431868	newly 5 weeks cumul	1008.	1688.72	{1541., 1939.}	67.5317	0.222801	newly 51 weeks cumul	1234.	2480.06	{2226., 2945.}	100.977	0.301298
31	newly 3 weeks spec	252.	285.52	{239., 332.}	15.8492	0.0625949	newly 3 weeks cumul	969.	1131.09	{991., 1277.}	17.2559	0.0674238	newly 5 weeks spec	111.	162.6	{128., 193.}	47.2072	0.162979	newly 5 weeks cumul	1242.	1508.29	{1290., 1716.}	21.8897	0.083847	newly 54 weeks cumul	1508.	1991.69	{1602., 2307.}	32.5391	0.119159
32	newly 3 weeks spec	205.	287.25	{243., 315.}	40.122	0.144869	newly 3 weeks cumul	891.	981.08	{866., 1044.}	11.1291	0.0455058	newly 5 weeks spec	111.	211.55	{172., 238.}	90.5856	0.276712	newly 5 weeks cumul	1156.	1439.76	{1238., 1541.}	24.5467	0.0941643	newly 46 weeks cumul	1578.	2502.36	{2035., 2734.}	58.5779	0.198112
33	newly 3 weeks spec	80.	241.3	{222., 260.}	201.625	0.478527	newly 3 weeks cumul	415.	982.63	{943., 1033.}	136.778	0.374071	newly 5 weeks spec	25.	134.	{119., 148.}	436.	0.727616	newly 5 weeks cumul	481.	1298.49	{1247., 1365.}	169.956	0.431022	newly 54 weeks cumul	518.	1686.74	{1623., 1767.}	225.625	0.512468
34	newly 3 weeks spec	107.	250.83	{159., 294.}	134.421	0.361205	newly 3 weeks cumul	518.	1037.65	{720., 1163.}	100.319	0.296695	newly 5 weeks spec	37.	137.36	{76., 170.}	271.243	0.552988	newly 5 weeks cumul	629.	1362.89	{898., 1537.}	116.676	0.32941	newly 56 weeks cumul	672.	1742.93	{1035., 2006.}	159.365	0.404403
35	newly 3 weeks spec	219.	303.36	{263., 345.}	38.5205	0.138977	newly 3 weeks cumul	914.	1119.15	{1011., 1226.}	22.6947	0.0876411	newly 5 weeks spec	143.	194.06	{158., 237.}	36.2378	0.129708	newly 5 weeks cumul	1246.	1557.33	{1393., 1745.}	25.2448	0.0961843	newly 51 weeks cumul	1672.	2285.	{1982., 2618.}	36.8134	0.133203
36	newly 3 weeks spec	304.	307.59	{279., 340.}	6.29276	0.0270542	newly 3 weeks cumul	1147.	1112.89	{1046., 1174.}	4.49608	0.0201083	newly 5 weeks spec	161.	203.5	{177., 228.}	26.4224	0.0999784	newly 5 weeks cumul	1520.	1567.04	{1493., 1643.}	4.66447	0.0197648	newly 49 weeks cumul	2076.	2380.53	{2220., 2503.}	14.8367	0.0593118
37	newly 3 weeks spec	209.	299.31	{251., 341.}	43.2105	0.153238	newly 3 weeks cumul	875.	1161.78	{1027., 1283.}	32.7749	0.121413	newly 5 weeks spec	117.	177.67	{141., 206.}	51.8547	0.17657	newly 5 weeks cumul	1151.	1575.69	{1372., 1755.}	36.8975	0.134308	newly 52 weeks cumul	1404.	2170.56	{1842., 2434.}	54.5983	0.186248
38	newly 3 weeks spec	55.	308.71	{285., 330.}	461.291	0.748439	newly 3 weeks cumul	284.	1263.5	{1213., 1313.}	344.894	0.648071	newly 5 weeks spec	18.	175.05	{155., 193.}	872.5	0.986479	newly 5 weeks cumul	331.	1672.76	{1612., 1732.}	405.366	0.70345	newly 55 weeks cumul	350.	2171.64	{2103., 2234.}	520.469	0.792596
39	newly 3 weeks spec	107.	271.03	{249., 298.}	153.299	0.401643	newly 3 weeks cumul	564.	1092.82	{1041., 1160.}	93.7624	0.286391	newly 5 weeks spec	42.	151.02	{131., 171.}	259.571	0.552706	newly 5 weeks cumul	669.	1444.29	{1367., 1531.}	115.888	0.333191	newly 55 weeks cumul	739.	1886.54	{1758., 2003.}	155.283	0.405619
40	newly 3 weeks spec	222.	329.83	{296., 366.}	48.5721	0.170628	newly 3 weeks cumul	944.	1243.23	{1163., 1306.}	31.6981	0.119036	newly 5 weeks spec	115.	206.07	{179., 233.}	79.1913	0.250879	newly 5 weeks cumul	1200.	1708.51	{1593., 1818.}	42.3758	0.152816	newly 52 weeks cumul	1524.	2428.97	{2234., 2643.}	59.3812	0.201527
41	newly 3 weeks spec	212.	287.23	{256., 313.}	35.4858	0.13003	newly 3 weeks cumul	904.	1071.45	{998., 1150.}	18.5232	0.073009	newly 5 weeks spec	89.	180.85	{154., 203.}	103.202	0.304838	newly 5 weeks cumul	1155.	1479.15	{1384., 1577.}	28.0649	0.106567	newly 51 weeks cumul	1447.	2107.82	{1954., 2249.}	45.6683	0.162052
42	newly 3 weeks spec	235.	240.67	{217., 262.}	6.06383	0.0257737	newly 3 weeks cumul	936.	863.34	{816., 901.}	8.03205	0.036629	newly 5 weeks spec	165.	169.14	{147., 191.}	8.13333	0.0346537	newly 5 weeks cumul	1287.	1236.22	{1171., 1292.}	4.6589	0.0209329	newly 46 weeks cumul	1777.	1980.5	{1858., 2098.}	11.8571	0.0481049
43	newly 3 weeks spec	116.	349.34	{310., 388.}	201.155	0.477044	newly 3 weeks cumul	596.	1394.73	{1277., 1477.}	134.015	0.368523	newly 5 weeks spec	40.	195.61	{170., 220.}	389.025	0.686956	newly 5 weeks cumul	704.	1853.3	{1703., 1958.}	163.253	0.419552	newly 56 weeks cumul	757.	2433.98	{2237., 2589.}	221.53	0.506096
44	newly 3 weeks spec	281.	314.01	{289., 344.}	12.6014	0.051036	newly 3 weeks cumul	1071.	1096.19	{1052., 1141.}	3.8422	0.0165227	newly 5 weeks spec	173.	225.84	{205., 246.}	30.5665	0.114544	newly 5 weeks cumul	1453.	1587.48	{1530., 1657.}	9.883	0.0408937	newly 47 weeks cumul	1985.	2611.89	{2479., 2781.}	31.5814	0.118533
45	newly 3 weeks spec	250.	306.22	{271., 342.}	22.528	0.0862871	newly 3 weeks cumul	978.	1150.54	{1073., 1230.}	17.6421	0.0697354	newly 5 weeks spec	142.	191.64	{164., 223.}	34.9577	0.127692	newly 5 weeks cumul	1328.	1588.34	{1484., 1712.}	19.6039	0.076815	newly 51 weeks cumul	1725.	2272.74	{2079., 2485.}	31.753	0.118404
46	newly 3 weeks spec	267.	300.79	{257., 339.}	13.9663	0.0554732	newly 3 weeks cumul	1078.	1089.18	{968., 1170.}	5.99443	0.0259789	newly 5 weeks spec	173.	200.48	{172., 226.}	17.4913	0.0687459	newly 5 weeks cumul	1493.	1538.31	{1345., 1653.}	6.93168	0.0295229	newly 49 weeks cumul	2102.	2362.71	{2000., 2589.}	13.8482	0.0555039
47	newly 3 weeks spec	255.	350.04	{309., 405.}	37.2706	0.135484	newly 3 weeks cumul	1074.	1268.02	{1159., 1389.}	18.0652	0.0710317	newly 5 weeks spec	129.	225.57	{192., 262.}	74.8605	0.239845	newly 5 weeks cumul	1366.	1776.72	{1625., 1960.}	30.0673	0.112921	newly 51 weeks cumul	1768.	2652.87	{2334., 3004.}	50.0492	0.174167
48	newly 3 weeks spec	231.	336.73	{301., 371.}	45.7706	0.162278	newly 3 weeks cumul	980.	1335.9	{1225., 1433.}	36.3163	0.13378	newly 5 weeks spec	109.	188.79	{163., 218.}	73.2018	0.23622	newly 5 weeks cumul	1224.	1777.91	{1628., 1907.}	45.2541	0.161363	newly 54 weeks cumul	1459.	2362.08	{2180., 2539.}	61.8972	0.208496
49	newly 3 weeks spec	157.	312.24	{287., 335.}	98.879	0.297809	newly 3 weeks cumul	774.	1262.37	{1222., 1307.}	63.0969	0.212291	newly 5 weeks spec	60.	176.19	{159., 191.}	193.65	0.466771	newly 5 weeks cumul	908.	1674.49	{1623., 1741.}	84.4152	0.265642	newly 55 weeks cumul	1013.	2189.76	{2124., 2265.}	116.166	0.334625
50	newly 3 weeks spec	221.	341.59	{318., 363.}	54.5656	0.188399	newly 3 weeks cumul	1020.	1321.94	{1290., 1357.}	29.602	0.112497	newly 5 weeks spec	108.	199.31	{180., 216.}	84.5463	0.265041	newly 5 weeks cumul	1290.	1786.11	{1746., 1828.}	38.4581	0.141224	newly 53 weeks cumul	1505.	2421.99	{2365., 2472.}	60.9296	0.206523
